# Genome-Wide Analysis Reveals Selective Modulation of microRNAs and mRNAs by Histone Deacetylase Inhibitor in B Cells Induced to Undergo Class-Switch DNA Recombination and Plasma Cell Differentiation

**DOI:** 10.3389/fimmu.2015.00627

**Published:** 2015-12-14

**Authors:** Tian Shen, Helia N. Sanchez, Hong Zan, Paolo Casali

**Affiliations:** ^1^Department of Microbiology and Immunology, University of Texas School of Medicine, UT Health Science Center, San Antonio, TX, USA

**Keywords:** AID, Blimp1, B cell, class-switch DNA recombination, epigenetics, HDAC, HDAC inhibitor, histone acetylation, microRNA, mRNA, mRNA-Seq, miRNA-Seq, plasma cell differentiation, somatic hypermutation

## Abstract

As we have suggested, epigenetic factors, such as microRNAs (miRNAs), can interact with genetic programs to regulate B cell functions, thereby informing antibody and autoantibody responses. We have shown that histone deacetylase (HDAC) inhibitors (HDI) inhibit the differentiation events critical to the maturation of the antibody response: class-switch DNA recombination (CSR), somatic hypermutation (SHM), and plasma cell differentiation, by modulating intrinsic B cell mechanisms. HDI repress the expression of AID and Blimp-1, which are critical for CSR/SHM and plasma cell differentiation, respectively, in mouse and human B cells by upregulating selected miRNAs that silenced *AICDA/Aicda* and *PRDM1/Prdm1* mRNAs, as demonstrated by multiple qRT-PCRs (*J Immunol* 193:5933–5950, 2014). To further define the selectivity of HDI-mediated modulation of miRNA and gene expression, we performed genome-wide miRNA-Seq and mRNA-Seq analysis in B cells stimulated by LPS plus IL-4 and treated with HDI or nil. Consistent with what we have shown using qRT-PCR, these HDI-treated B cells displayed reduced expression of *Aicda* and *Prdm1*, and increased expression of miR-155, miR-181b, and miR-361, which target *Aicda*, and miR-23b, miR-30a, and miR-125b, which target *Prdm1*. In B cells induced to undergo CSR and plasma cell differentiation, about 23% of over 22,000 mRNAs analyzed were expressed at a significantly high copy number (more than 20 copies/cell). Only 18 (0.36%) of these highly expressed mRNAs, including *Aicda*, *Prdm1*, and *Xbp1*, were downregulated by HDI by 50% or more. Further, only 16 (0.30%) of the highly expressed mRNAs were upregulated (more than twofold) by HDI. The selectivity of HDI-mediated modulation of gene expression was emphasized by unchanged expression of the genes that are involved in regulation, targeting, or DNA repair processes of CSR, as well as unchanged expression of the genes encoding epigenetic regulators and factors that are important for cell signaling or apoptosis. Our findings indicate that, in B cells induced to undergo CSR and plasma cell differentiation, HDI modulate selected miRNAs and mRNAs, possibly as a result of HDACs existing in unique contexts of HDAC/cofactor complexes, as occurring in B lymphocytes, particularly when in an activated state.

## Introduction

Epigenetic markers or factors, such as DNA methylation, histone posttranslational modifications, and microRNAs (miRNAs), dynamically regulate gene activities. As we have contended, epigenetic markers/factors “interact” with genetic programs to regulate B cell functions, such as class-switch DNA recombination (CSR), somatic hypermutation (SHM), and differentiation to memory B cell or plasma cell, thereby informing antibody and autoantibody responses (1). CSR and SHM are B cell-intrinsic differentiation processes that underpin the generation of class-switched and high-affinity antibodies, such as those that clear microbial pathogens or kill tumor cells. CSR and SHM critically require activation-induced cytidine deaminase (AID, encoded by *AICDA* in humans and *Aicda* in mice), which is specifically and highly induced in B cells in both T-dependent and T-independent antibody responses (2). Class-switched and hypermutated B cells further differentiate into antibody-secreting plasma cells in a fashion critically dependent on B lymphocyte-induced maturation protein 1 (Blimp1, encoded by *PRDM1* in human beings and *Prdm1* in mice) (3), or transition to long-lived memory B cells, which can differentiate into plasma cells upon reactivation by antigen to mediate an anamnestic response (4). Pathogenic autoantibodies, including those to nuclear components in systemic lupus erythematosus (SLE) patients (5, 6), are also class-switched and hypermutated (7, 8). Thus, epigenetic dysregulation of B cells can result in aberrant antibody responses to exogenous antigens, such as those on viruses and bacteria, or self-antigens, such as chromatin, histones, and dsDNA in lupus (1, 7).

The chromatin structure is comprised of DNA and histones. The basic repeating unit of chromatin is the nucleosome, a 147 bp of DNA chain wrapped around one histone octamer composed of two copies of each of four histones: H2A, H2B, H3, and H4. Histone posttranslational modifications include phosphorylation of serine or threonine residues, methylation of lysine or arginine, acetylation and deacetylation of lysines, and ubiquitylation and sumoylation of lysines. All these posttranslational modifications play an important role in regulating gene expression (9, 10). Histone acetylation and deacetylation, which are essential for gene regulation, are typically modulated by histone acetyltransferase (HAT) and histone deacetylase (HDAC) (9, 10). Histone acetylation catalyzed by HAT will result in a loose chromatin structure, which enables DNA binding proteins to activate gene transcription, while histone deacetylation catalyzed by HDAC will result in a condensed chromatin structure, which prevents binding of transcription factors or proteins to DNA and silence gene expression. HDAC inhibitors (HDI) alter gene expression by altering chromatin accessibility (11, 12).

MicroRNAs also play an important role in regulation of the genes involved in CSR, SHM, and plasma cell differentiation (1, 7, 13). miRNAs are small (~22 nucleotides), evolutionarily conserved non-coding RNAs derived from much larger primary transcripts encoded by their “host genes.” miRNAs bind to complementary sequences within the 3′ untranslated region (3′ UTR) of their target mRNAs and negatively regulate protein expression at the posttranscriptional level through inhibition of translation and/or reduction of mRNA stability (14, 15). The mammalian genome encodes thousands of miRNAs that collectively affect the expression of more than half of protein-coding genes. In addition, miRNAs have been implicated as fine-tuning regulators controlling diverse biological processes at posttranscriptional level. They can potentially regulate every aspect of cellular activity, from proliferation and differentiation to apoptosis, as well as modulate a large range of physiological and pathological processes. miRNAs likely play important roles in B cell development and peripheral differentiation, as well as T cell stage-specific differentiation and autoimmunity. Some miRNAs, including miR-155, miR-181b, and miR-361, can silence AID expression, whereas miR-30a and miR-125b can silence Blimp-1 expression (16). These miRNAs bind to evolutionarily conserved miRNA target sites in the 3′ UTR of *Aicda* and *Prdm1* mRNAs and cause degradation of the mRNA transcripts and/or inhibit their translation.

We have recently shown that HDI, such as short-chain fatty acid valproic acid and butyrate, inhibit the expression of AID and Blimp-1 in human and mouse B cells *in vivo* and *in vitro* and regulate intrinsic B cell functions that are critical in shaping effective antibody and autoantibody responses (16). Valproic acid or sodium valproate (VPA, 2-propyl-pentanoic acid sodium) is widely used to treat epilepsy and mood disorders. VPA can selectively inhibits class I HDACs, particularly, HDAC1 and HDAC2, and less effectively, class IIa HDACs among the four HDAC classes identified in mammals (17, 18) to alter gene expression by changing chromatin accessibility. We have further shown that HDI, such as VPA and butyrate, inhibit AID and Blimp1 expression by upregulating miR-155, miR-181b, and miR-361, which silenced *AICDA/Aicda* mRNA, and miR-23b, miR-30a, and miR-125b, which silenced *PRDM1/Prdm1* mRNA (16). The selectivity of HDI-mediated silencing of *AICDA/Aicda* and *PRDM1/Prdm1* was emphasized by unchanged expression of HoxC4 and Irf4 (important inducers/modulators of *AICDA/Aicda*), Rev1 and Ung (central elements for CSR/SHM), and Bcl6, Bach2, or Pax5 (repressors of *PRDM1/Prdm1* expression), as well as unchanged expression of miR-19a/b, miR-20a, and miR-25, which are not known to regulate *AICDA/Aicda* or *PRDM1/Prdm1*. Epigenetic modulations always display a cell type- and cell stage-specific regulation pattern of gene expression (19). To extend our findings and further define the selectivity of HDI-mediated modulation of miRNAs and gene expression, we performed genome-wide miRNA-Seq and mRNA-Seq analysis in B cells induced to undergo CSR and plasma cell differentiation in the presence of VPA. Here, we showed that this HDI modulated selected miRNAs and mRNAs, possibly as a result of HDACs existing in unique contexts of HDAC/cofactor.

## Materials and Methods

### Stimulation of Mouse B Cells for CSR and Plasma Cell Differentiation, and HDI Treatment

C57BL/6 mice were purchased from The Jackson Laboratory and maintained at the University of Texas Health Science Center at San Antonio (UTHSCSA) animal facility. The Institutional Animal Care and Use Committee of UTHSCSA approved all animal protocols. Naïve IgD^+^ B cells were isolated from 8-week-old C57BL/6 mice as described (16, 20). B cells were resuspended in RPMI 1640 medium with 10% FBS, 50 mM β-mercaptoethanol, and 1× antibiotic–antimycotic mixture (15240-062; Invitrogen) (FBS-RPMI) at 37°C and stimulated with LPS (3 μg/ml) from *Escherichia coli* (055:B5; Sigma-Aldrich) plus IL-4 (5 ng/ml; R&D Systems) for CSR to IgG1/IgE and plasma cell differentiation. HDI [VPA 500 μM, a concentration comparable to serum concentration of VPA-treated mice (21)] or nil were also added to the cultures. Cells were collected 60 h later for qRT-PCR, mRNA-Seq, and miRNA-Seq, or 96 h later for surface Ig analysis by flow cytometry (16, 22).

### RNA Extraction and High Throughput mRNA-Seq and miRNA-Seq

Total RNA was extracted from 2 × 10^6^ cells using miRNeasy^®^ Mini Kit (Qiagen), as previously described (16). RNA integrity was verified using an Agilent Bioanalyzer 2100 (Agilent). Next generation mRNA-Seq and small RNA-Seq were performed by the Genome Sequencing Facility (Greehey Children’s Cancer Research Institute, GCCRI), UTHSCSA. High-quality RNA (RNA Integrity number or RIN.9.0) was processed using an Illumina TruSeq RNA sample prep kit v2 or TruSeq Small RNA Sample Prep kit following the manufacturer’s instructions (Illumina). Clusters were generated using TruSeq Single-Read Cluster Gen. Kit v3-cBot-HS on an Illumina cBot Cluster Generation Station. After quality control procedures, individual mRNA-Seq or small RNA-Seq libraries were then pooled based on their respective 6-bp index portion of the TruSeq adapters and sequenced at 50 bp/sequence, read using an Illumina HiSeq 2000 sequencer. The barcode combinations were further crosschecked by Illumina Experiment Manager software. Sequence data were checked by assurance (QA) pipeline and initial genome alignment (Alignment). Approximately 33 million and 5 million reads per sample were generated in mRNA-Seq and miRNA-Seq, respectively. After the sequencing run, demultiplexing with CASAVA was employed to generate the fastq file for each sample. All sequencing reads were aligned with their reference genome (UCSC mouse genome build mm9) using TopHat2 default settings and the Bam files from alignment were processed using HTSeq-count to obtain the counts per gene in all samples. Quality control statistical analysis of outliers, intergroup variability, distribution levels, PCA, and hierarchical clustering analysis were performed for statistical validation of the experimental data.

### Quantitative RT-PCR (qRT-PCR) of mRNAs and miRNAs

For mRNA quantification, post-recombination Iμ-C_H_ and mature V_H_DJ_H_-C_H_ transcripts. cDNA was synthesized from total RNA with the SuperScript™ III First-Strand Synthesis System (Invitrogen) using oligo-dT primer. Transcript expression was measured by qRT-PCR using the appropriate primers, as previously reported (16) using a Bio-Rad MyiQ™ Real-Time PCR Detection System (Bio-Rad Laboratories) to measure SYBR Green (IQ™ SYBR^®^ Green Supermix, Bio-Rad Laboratories) incorporation with the following protocol: 95°C for 15 s, 40 cycles of 94°C for 10 s, 60°C for 30 s, 72°C for 30 s. Data acquisition was performed during the 72°C extension step. Melting curve analysis was performed from 72 to 95°C. For quantification of mature miRNA transcripts, RNA was extracted from 0.2–5 × 10^6^ cells using miRNeasy^®^ Mini Kit (Qiagen) and then reverse-transcribed with miScript II RT Kit (Qiagen) using the miScript HiSpec buffer. A Bio-Rad MyiQ™ Real-Time PCR Detection System was used to measure SYBR Green (miScript SYBR Green PCR Kit; Qiagen) incorporation according to manufacturer’s instructions. Mature miRNA forward primers were used at 250 nM in conjunction with the Qiagen miScript Universal Primer and normalized to expression of small nuclear/nucleolar RNAs Rnu6/RNU61/2, Snord61/SNORD61, Snord68/SNORD68, and Snord70/SNORD70. The ΔΔCt method was used for qRT-PCR data analysis with Microsoft Excel.

### Statistical Analysis

Statistical analysis was performed to determine *p* values by paired and unpaired Student’s *t-*test, and *p* values <0.05 were considered significant.

## Results

### HDI Inhibit CSR and Plasma Cell Differentiation

**Figure 1:**
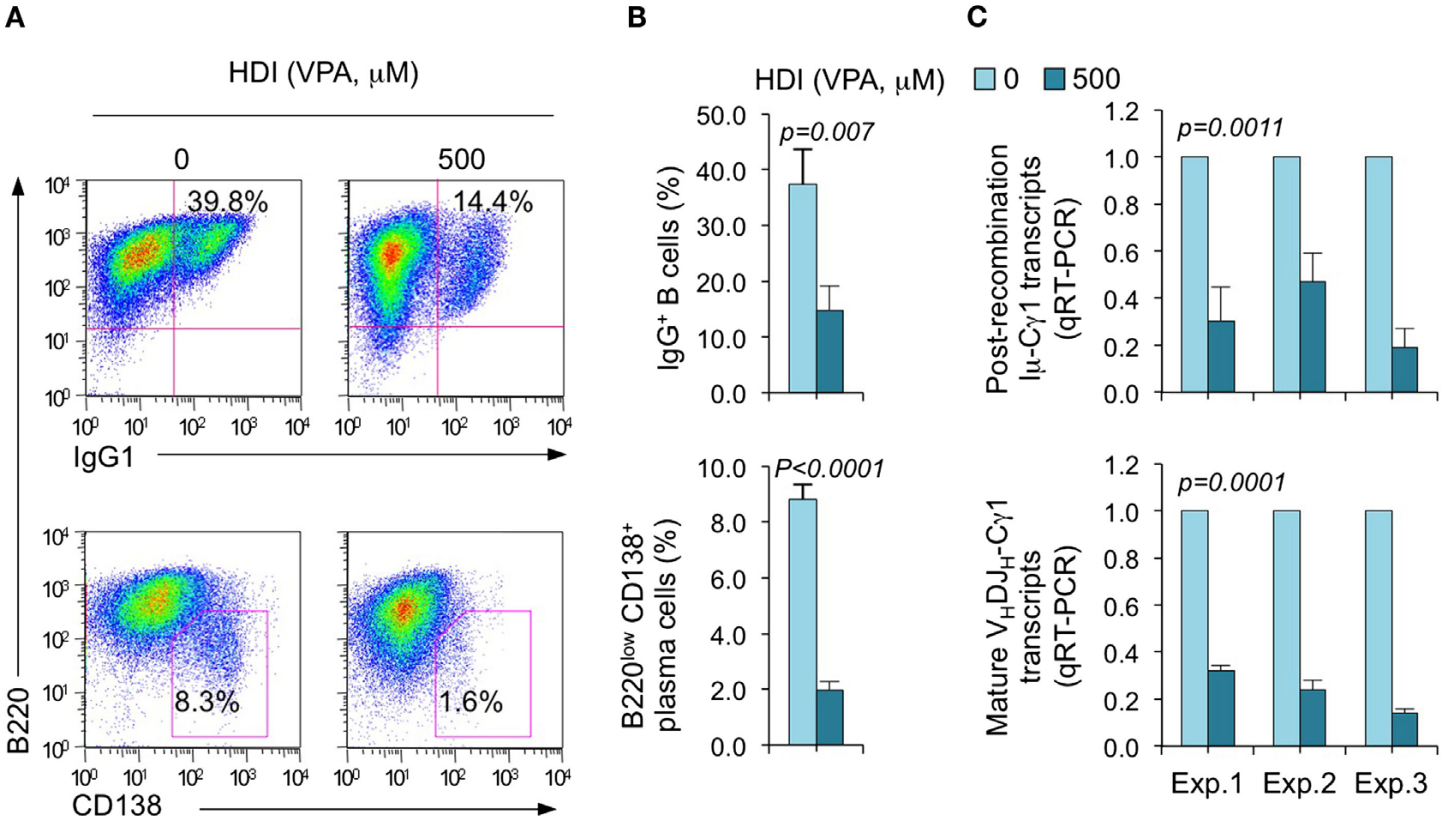
**HDI inhibits B cell CSR and plasma cell differentiation**. Purified spleen B cells were stimulated with LPS plus IL-4 in the presence of HDI (VPA, 500 μM) or nil. **(A)** Surface expression of B220 and IgG1 was measured by flow cytometry 96 h after the stimulation, Data are one representative of three independent experiments. **(B)** The average percentage of IgG1^+^ B cells and B220^low^ CD138^+^ plasma cell in nil or HDI-treated cells in the three experiments. **(C)** Mature V_H_DJ_H_-Cγ1 transcripts and post-recombination Iμ-Cγ1 transcripts, both hallmarks of completed CSR, were analyzed by qRT-PCR and normalized to *Cd79b* transcripts in B cells stimulated for 60 hours, measured by qRT-PCR and normalized to *Cd79b* transcripts. Data are from three independent experiments. *p* values, paired *t*-test.

We have shown that HDI repress the expression of AID and Blimp-1, which are critical for CSR/SHM and plasma cell differentiation, respectively, in mouse and human B cells by upregulating selected miRNAs that silenced *AICDA/Aicda* and *PRDM1/Prdm1* mRNAs, as demonstrated by multiple qRT-PCRs (16). To further define the selectivity of HDI-mediated modulation of miRNA and gene expression, we stimulated purified mouse B cells with LPS plus IL-4, which induce B cells to undergo CSR to IgG1 or IgE and differentiate to plasma cells in the presence of HDI (VPA, 500 μM) or nil. Consistent with our previous findings (16), HDI significantly inhibited CSR and plasma cell differentiation, as shown by greatly reduced surface IgG1^+^ B cells and B220^low^ CD138^+^ plasma cells (Figure 1). HDI inhibition of CSR was further confirmed by decreased numbers of post-recombination Iμ-Cγ1 and mature V_H_DJ_H_-Cγ1 transcripts.

### HDI-Mediated Modulation of mRNA Expression in B Cells is Highly Selective

**Figure 2:**
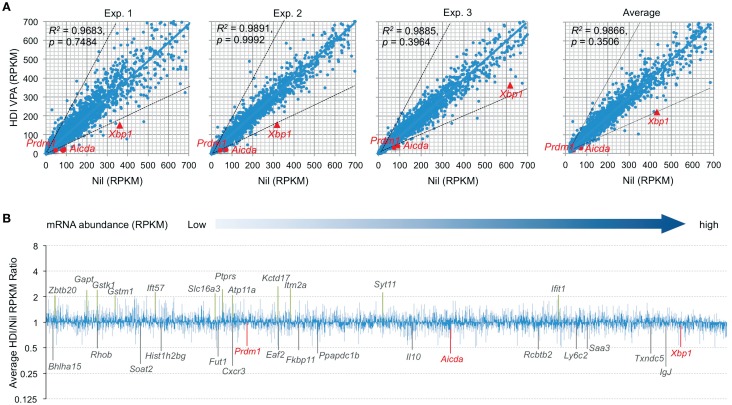
**HDI selectively inhibits mRNA expression in B cells induced to undergo CSR and plasma cell differentiation**. B cells were cultured with LPS plus IL-4 in the presence of HDI (VPA, 500 μM) or nil for 60 h before preparation of RNA for mRNA-Seq. **(A)** Scatter plots of average mRNA expression levels in three independent experiments (reads per kilobase per million mapped reads, RPKMs), in LPS plus IL-4-stimulated B cells treated with VPA versus those in LPS plus IL-4-stimulated B cells treated with nil. Each plot corresponds to one individual mRNA expression level. **(B)** Bar graphs depict the average folds of changes of mRNA expression levels (average RPKMs from three independent experiments) in LPS plus IL-4-stimulated B cells treated with HDI, as compared to those in B cells treated with nil. Only the mRNAs at average RPKM >20 in LPS plus IL-4-stimulated B cells treated with nil are included. *Aicda, Prdm1*, and *Xbp1* mRNAs and the mRNAs that were increase or decreased or increased in average by more than twofold are indicated. *p* values, paired *t*-test.

Histone deacetylases remove the acetyl groups from the histone lysine residues leading to the formation of a condensed and transcriptionally silenced chromatin. HDI block this activity, thereby increasing histone acetylation and alteration of gene expression. It has been shown that HDI, such as TSA, suberoylanilide hydroxamic acid (SAHA), MS-275, and FK228, could alter the expression of 5–20% of genes (23). To further define the modulation of gene expression by HDI in B cells that undergo CSR and plasma cell differentiation, we performed high throughput mRNA-Seq to analyze the transcriptome in B cells stimulated by LPS plus IL-4 and treated with HDI (VPA, 500 μM) or nil. In general, one RPKM (read per kb per million reads) of mRNA represents approximately one copy of transcript per cell (24). In three independent experiments, HDI did not significantly alter overall mRNA expression (*p* = 0.99925, 0.74835, and 0.39640, respectively), although the average PRKM was slightly reduced from 42.71 to 38.49 (*p* = 0.3506) (Figure 2). Among over 22,000 genes analyzed, about 5,000 of them were significantly expressed (more than 20 RPKM, average more than 20 transcripts per cells) in B cells stimulated by LPS plus IL-4. Upon treatment with HDI, only 18 (0.36%) of the “highly” expressed genes, including *Aicda, Prdm1, Xbp1, Bhlha15, RRhob, Soat2, Hist1h2bg, Fut1, Cxcr3, Eaf2, Fkbp11, Ppapdc1b, Il10, Rcbtb2, Ly6c2, Saa3, Txndc5*, and *IgJ*, were downregulated, on average, by 50% or more. The mRNA of Cxcr3 (C-X-C motif chemokine receptor 3), which is highly expressed in IgG1^+^ memory B cells and can promote the production of IgG1 autoantibodies (25, 26), was reduced by about 70% by HDI. The mRNA of Saa3 (Serum amyloid A3), which can interact with Tlr4 and induce Tlr4-mediated NF-κB activation (27), was reduced by over 51%. Sixteen (0.30%) of the “highly” expressed (more than 20 RPKM) genes, including *Gstk1, Gstm1, Zbtb20, Gapt, Ift57, Slc16a3, Ptprs, Atp11a, Kctd17, Itm2a, Syt11*, and *Ifit1*, were upregulated by HDI by more than twofold (Figures 2A,B). Downregulation or upregulation of the above genes by more than twofold was consistent in all the three experiments, suggesting that the modulation of gene expression by HDI is highly selective.

### *Aicda, Prdm1*, and *Xbp1* are Selectively Silenced by HDI

**Figure 3:**
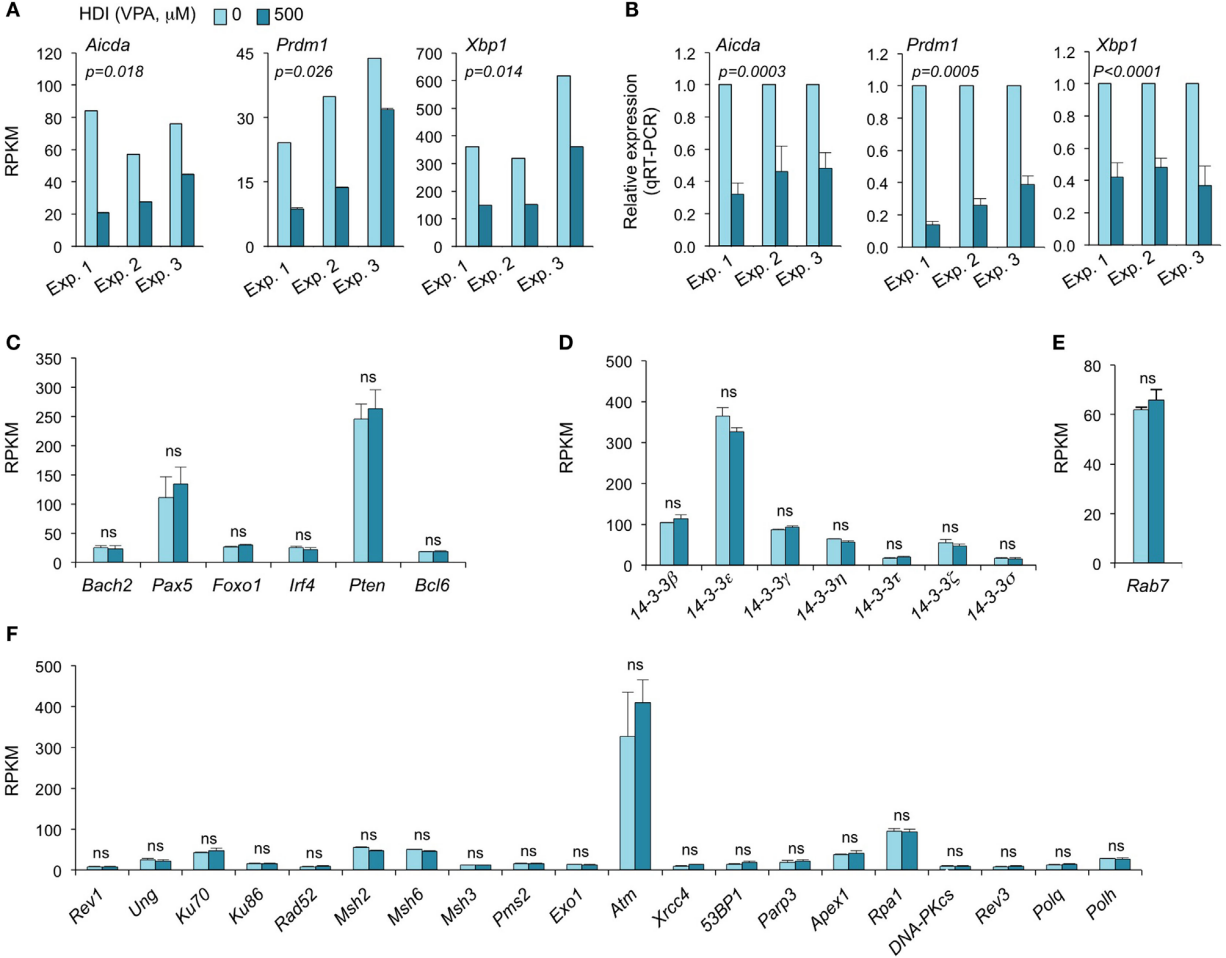
**HDI inhibits the expression of *Aicda*, *Prdm1*, and *Xbp1*, but not other elements that are important for CSR/SHM and plasma cell differentiation**. B cells were stimulated with LPS plus IL-4 for 60 h, in the presence of HDI (VPA, 500 μM) or nil. **(A)** Expressions of *Aicda, Prdm1*, and *Xbp1* were mRNAs analyzed by mRNA-Seq. Data are from three independent experiments. **(B)** qRT-PCR validation of *Aicda, Prdm1*, and *Xbp1* expressions. Data are from three independent experiments. *p* values, paired *t*-test. Expressions of **(C)** *Bach2, Pax5, Foxo1, Irf4, Pten*, and *Bcl6*, **(D)** the seven 14-3-3 isoforms, **(E)** Rab7, and **(F)** the genes that encode the proteins that have been shown to play important roles in CSR and/or SHM were analyzed by mRNA-Seq and depicted as RPKM. Data are from three independent experiments. ns, not significant, paired *t*-test.

Consistent with our real-time qRT-PCR results (16), the mRNA-Seq experiments further demonstrated that *Aicda*, *Prdm1*, and *Xbp1* transcripts were significantly downregulated by HDI (Figures 2 and 3A–C). In all three independent experiments, *Aicda*, *Prdm1*, and *Xbp1* were consistently reduced by HDI by more than 57, 48, and 47%, respectively, ranking 7th, 21st, and 24th, of the most downregulated genes among the total of more than 5,000 genes that were highly expressed (more than 20 RPKM) in B cells stimulated by LPS plus IL-4. Thus, these deep sequencing experiments showed that *Aicda*, *Prdm1*, and *Xbp1* are selectively inhibited by HDI in B cells undergoing CSR and plasma cell differentiation.

### HDI Does Not Alter the Expression of 14-3-3 Adaptors or Rab7

The 14-3-3 adaptor family consists of a class of phosphoserine/phosphothreonine (pSer/Thr)-binding proteins, which include seven isoforms (14-3-3β, 14-3-3ε, 14-3-3γ, 14-3-3η, 14-3-3σ, 14-3-3τ, and 14-3-3ζ) encoded by different genes. 14-3-3 proteins are involved in a variety of cellular processes, including gene regulation, differentiation, and cell cycle progression (28). As we have shown, 14-3-3 adaptors play an important role in targeting the CSR machinery to S regions by virtue of their ability to bridge proteins with DNA or other proteins (29, 30). They directly interact with AID, protein kinase A catalytic subunit-α (PKA-Cα), regulatory inhibitory subunit-α (PKA-RIα), and uracil DNA glycosylase (Ung) to function as scaffolds to stabilize these enzymatic CSR elements on S regions. 14-3-3 expression has been suggested to be regulated by posttranscriptional modulation. 14-3-3 can interact with the phosphorylation sites of HDAC4, 5, and 7 and regulate cellular localization of these HDACs (31). As we have shown, the expression of most 14-3-3 proteins is significantly upregulated by the induction of CSR in B cells (2, 29, 30, 32). To determine whether potential alterations of 14-3-3 proteins in B cells could contribute to the HDI-mediated inhibition of CSR, we analyzed our mRNA-Seq data on the seven 14-3-3 isoforms. None of the seven 14-3-3 isoforms were significantly altered by HDI in B cells stimulated by LPS plus IL-4 (Figure 3D). 14-3-3ζ expression can be modulated by miR-193b and miR-375, which target 3′ UTR of 14-3-3ζ mRNA, in cancer cells (33, 34). However, these two miRNAs were not expressed in LPS plus IL-4-stimulated B cells (not shown).

We have recently demonstrated that Rab7, a small GTPase, plays an important role in CSR, through activation of the canonical NF-κB pathway and induction of AID expression (35). Like 14-3-3 adaptors, Rab7 expression is upregulated by the stimuli that induce CSR in B cells. To analyze whether the HDI-mediated downregulation of AID was at least partially due to a potential alteration of Rab7 expression, we analyzed Rab7 mRNA levels in B cells treated with HDI or nil. As shown by mRNA-Seq, HDI did not significantly alter Rab7 expression (Figure 3E). In one of the three experiments, Rab7 mRNA levels were virtually the same in B cells treated with HDI or nil. In the other two experiments, Rab7 mRNA was even slightly (*p* = 0.20) increased by HDI. Thus, downregulation of the AID-targeting 14-3-3 adaptors or downregulation of the CSR-regulating Rab7 small GTPase play no role in HDI-mediated modulation of CSR.

### HDI Does Not Alter the Expression of DNA Repair Factors that Are Important for CSR and SHM

Class-switch DNA recombination and SHM are tightly regulated and both are effected by a two-step process: (i) DNA lesions initiated by AID and (ii) lesion repair by the combined intervention of DNA replication and repair factors (2, 36, 37). Many DNA repair factors, including the base excision repair factor Ung, mismatch repair factors Msh2, Msh3, Msh6, Pms2, and Exo1 nuclease, translesion synthesis (TLS) DNA polymerases Rev3, Rev1, Polθ, and Polη, as well as DSB repair factors Ku70/Ku80, Rad52, RPA, and DNA-PK play important roles in CSR and/or SHM (2, 37–41). Dysregulation of these DNA repair factors can result in altered CSR/SHM, and thereby the antibody response. To define whether the HDI-mediated reduction of CSR and plasma cell differentiation was associated with any alteration in these factors, we analyzed the mRNA-Seq data for the expression of these factors. None of them was significantly altered by HDI (Figure 3F).

### HDI Does Not Significantly Alter the Expression of Epigenetic Regulators HATs, HDACs and Tet Proteins

**Figure 4:**
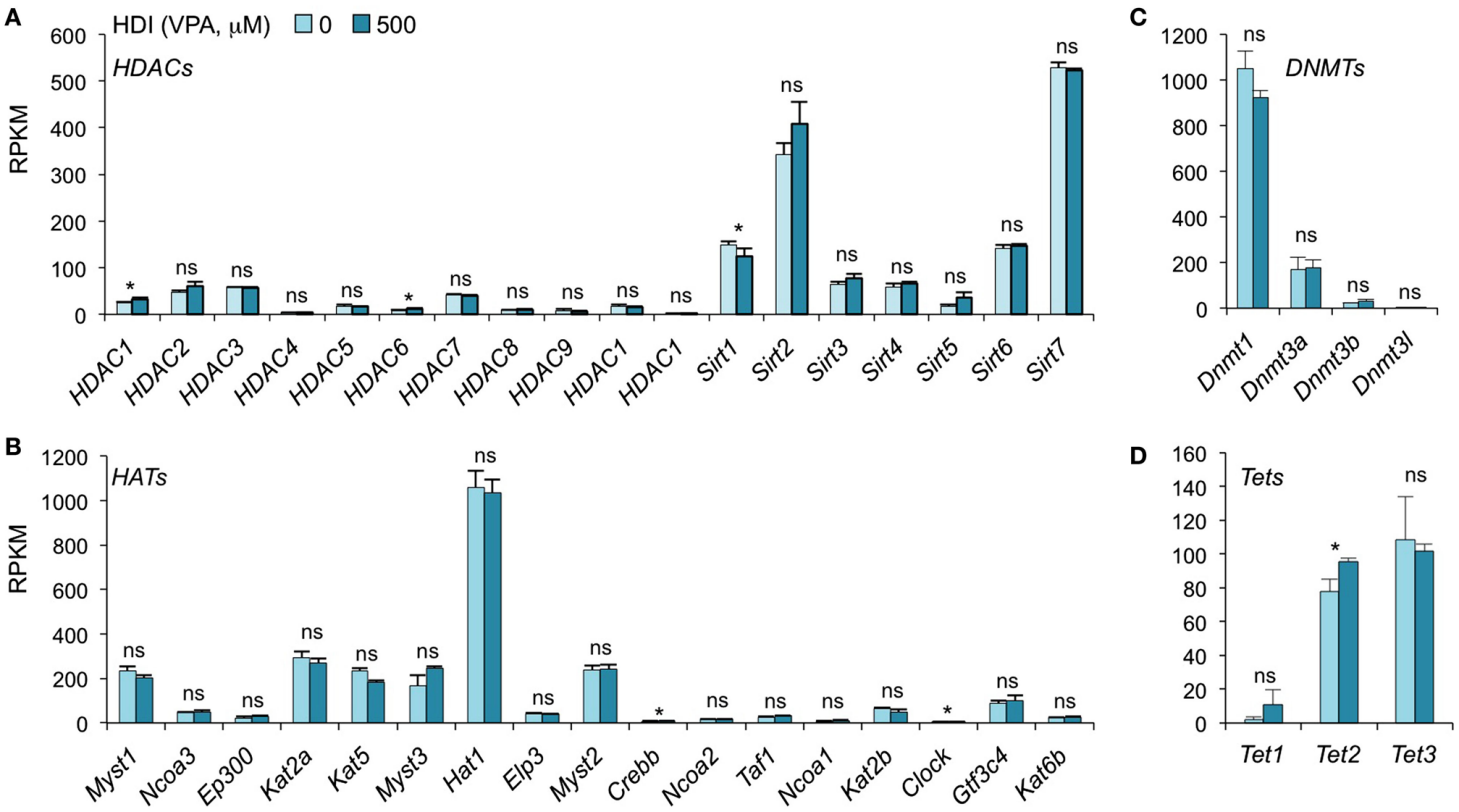
**HDI does not alter the expression of the epigenetic regulators HDACs, HATs, DNMTs, and Tet proteins**. B cells were stimulated with LPS plus IL-4 for 60 h, in the presence of HDI (VPA, 500 μM) or nil. mRNA expressions of the genes that encode **(A)** HDACs and **(B)** HATs, which modulate histone acetylation, and **(C)** DNMTs, as well as **(D)** Tet1, Tet2, and Tet3, which modulate DNA methylation, were analyzed by mRNA-Seq and depicted as RPKM. Data are from three independent experiments. *p < 0.05; ns, not significant, paired *t*-test.

In addition to inhibiting catalytic activity of HDACs, HDI have been suggested to selectively change the expression of some epigenetic regulators in certain type of cells. SAHA, a pan HDAC inhibitor, has been shown to downregulate HDAC7 expression in fibroblast cell lines (42). MS-275, TSA, and VPA downregulate DNMT1 protein expression in testis and embryonal carcinoma, as butyrate, SAHA, and PD98059 do in LNCaP prostate cancer cells (43). To determine whether HDI alter the expression of HDACs or DNMTs, as well as HATs and Tet1/Tet2/Tet3, which also mediate histone acetylation and DNA methylation, respectively, we analyzed the mRNA levels of these genes in our mRNA-Seq data (Figure 4). Thirty-six out of the 42 gene transcripts analyzed were not significantly altered by HDI, while *HDAC1, HDAC6, Clock*, and *Tet2* were marginally increased (by 24.63, 36.64, 9.57, and 23.19%, respectively), and *Sirt1* and *Crebbp* were slightly reduced (by 16.54 and 10.11%, respectively) by HDI. Thus, epigenetic regulator genes are not significantly modulated by HDI in B cells undergoing CSR and plasma cell differentiation.

### HDI Does Not Alter the Genes that Are Important in Cell Apoptosis

**Figure 5:**
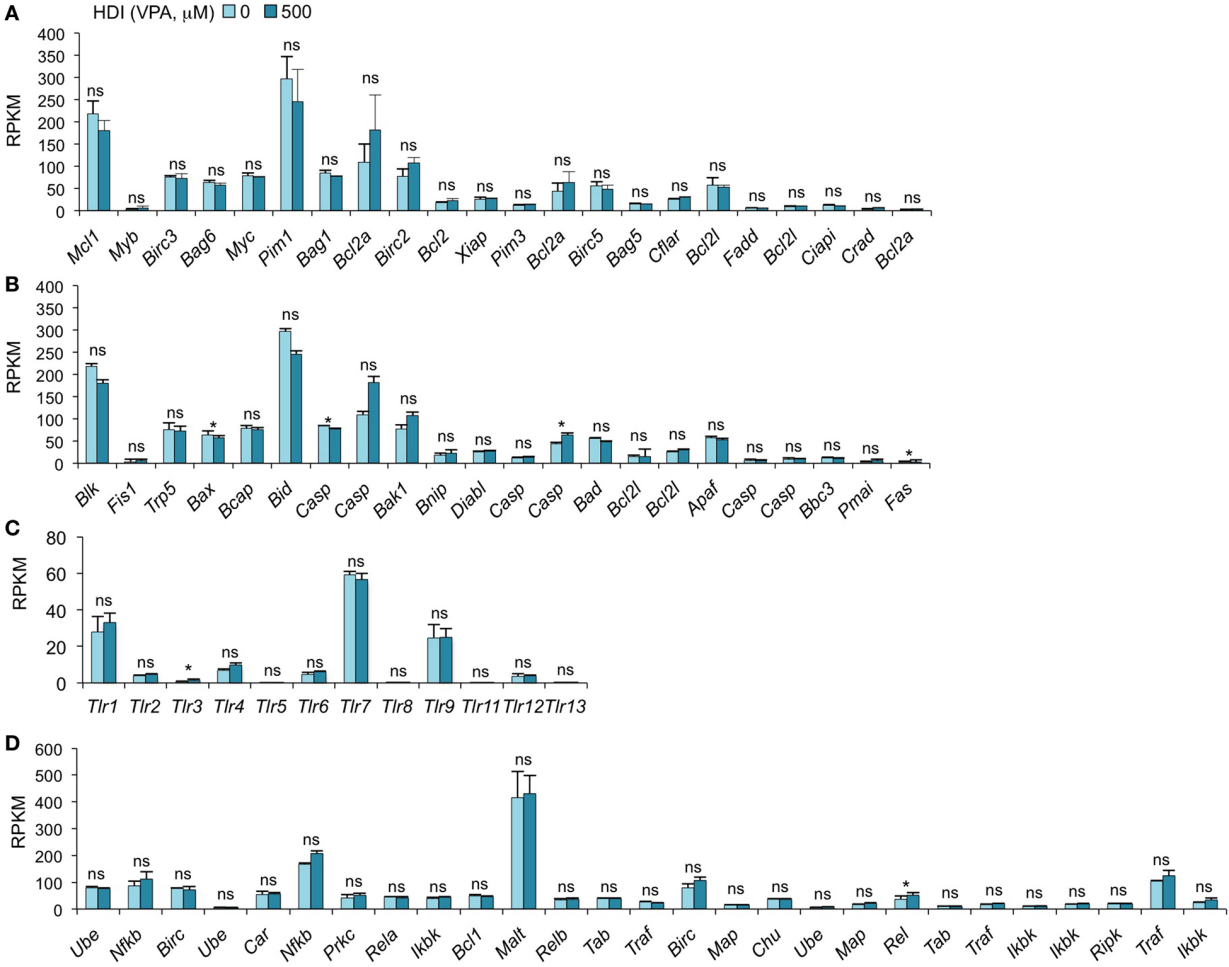
**HDI does not significantly alter the expression of the genes that are important for apoptosis, the genes that encode TLRs and the genes that are involved in NF-κB signaling**. B cells were stimulated with LPS plus IL-4 for 60 h, in the presence of HDI (VPA, 500 μM) or nil. mRNA expressions of the genes that encode **(A)** anti-apoptotic factors, **(B)** pro-apoptotic factors, **(C)** Toll-like receptors, and **(D)** the factors that are involved in NF-κB-signaling pathway were analyzed by mRNA-Seq and depicted as RPKM. Data are from three independent experiments. *p < 0.05; ns, not significant, paired *t*-test.

We have recently found that HDI inhibit, in a dose-dependent fashion, CSR and plasma cell differentiation without altering B cell proliferation or B cell and plasma cell viability (16). We have shown by qRT-PCR that the expression of the anti-apoptotic genes *Bcl2*, *Mcl1*, and *Bcl2l1*, which enhance B cell and plasma cell survival, was unaltered or increased by HDI *in vivo* and *in vitro* (16). Consistent with these findings, our mRNA-Seq data show that the expressions of all these genes were not altered by HDI (*p* = 0.28, 0.21, or 0.27) (Figure 5A). In addition, other 19 anti-apoptotic genes and 22 pro-apoptotic genes were also unchanged by HDI (Figures 5A,B). Thus, these findings further demonstrate that HDI significantly reduce CSR and plasma cell differentiation, without altering cell viability.

### HDI Does Not Significantly Alter TLR Expression and the Genes Involved in NF-κB Signaling

Toll-like receptors (TLRs) are a family of conserved pattern recognition receptors that sense diverse types of microbe-associated molecular patterns (MAMPs). Engagement of B cell TLRs by MAMPs not only induces T-independent antibody responses but also plays an important role in the early stages of T-dependent antibody responses, before specific T cell help becomes available (44). We have shown that TLR1/2-, TLR4-, TLR7-, or TLR9-signaling synergizes with BCR-signaling, which enhances TLR-dependent activation of the canonical NF-κB pathway, to induce AID and enable CSR (20). Given the important role of TLR-signaling and NF-κB pathway in the induction of AID, an alteration in the expression of TLR and the factors that are involved in NF-κB pathway would result in a change of AID expression and CSR. As shown by mRNA-Seq, the expression of 13 TLRs and 27 genes that are involved in NF-κB-signaling pathway was not altered by HDI (Figures 5C,D).

### HDI Inhibits Expression of Selected mRNAs in B Cells

**Figure 6:**
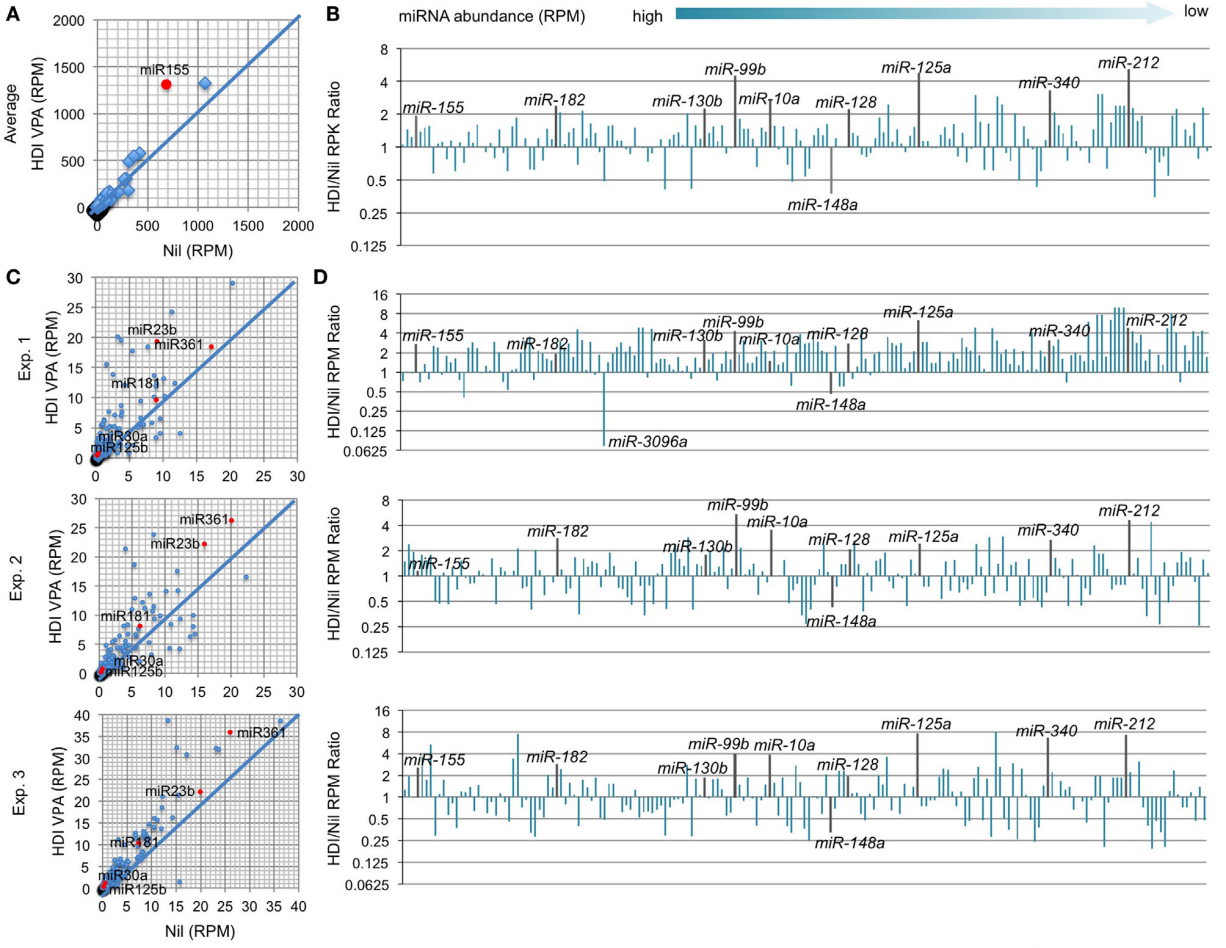
**HDI selectively inhibits miRNA expression in B cells induced to undergo CSR and plasma cell differentiation**. B cells were cultured with LPS plus IL-4 in the presence of HDI (VPA, 500 μM) or nil for 60 h before preparation of RNA for miRNA-Seq. **(A)** Scatter plots of average (in three independent experiments) miRNA expression levels (RPMs) in B cells treated with HDI versus that in B cells treated with nil. *P* values, paired *t*-test. **(B)** Bar graphs depict the changes of folds of the average miRNA expression levels (RPMs) in B cells treated with HDI as compared to that in B cells treated with nil. **(C)** Bar graphs depict the changes of folds of miRNA expression levels (RPMs) in B cells treated with HDI as compared to that in B cells treated with nil, in three independent experiments. **(D)** Scatter plots of miRNA expression levels in B cells treated with HDI versus those in B cells treated with nil, in three independent experiments. Only the miRNAs at average RPM > 0.5 in LPS plus IL-4-stimulated B cells treated with nil are included.

In B cells induced to undergo CSR and plasma cell differentiation, the number of genes that were downregulated by HDI nearly equated that of genes that were upregulated by HDI (Figure 2), suggesting that HDI can modulate gene expression by a mechanism other than directly increasing histone acetylation. Indeed, generally, only a small number of genes are thought to be directly modulated by changes in histone acetylation. Thus, it is possible that HDI upregulate the expression of genes, which negatively regulate the expression of other genes. HDI can modulate the expression of miRNAs, which silence target mRNAs by inducing their degradation and/or reducing their translation. We have recently shown that HDI downregulated the expression of AID and Blimp-1 by upregulating miR-155, miR-181b, and miR-361, which silence *Aicda* mRNA, and miR-23b, miR-30a, and miR-125b, which silence *Prdm1* mRNA, but not miR-19a/b, miR-20a, and miR-25, which are not known to regulate *Aicda, Prdm1*, or *Xbp1* (16). To further define the modulation of miRNA by HDI, we analyzed microRNAome in LPS and IL-4 stimulated B cells treated with HDI or nil by miRNA-Seq (Figure 6). In stimulated B cells, the average RPM of 520 miRNAs analyzed was 26.9 (10.75, if one excludes the three most abundant miRNAs miR-21a, miR-191, and miR-146a). Upon HDI treatment, the average RPM of all the miRNAs were slightly increased to 32.19 (13.63, if we exclude the three most abundant miRNAs miR-21a, miR-191, and miR-146a) (*R*^2^ = 0.9811, *p* = 0.5134). A total of 185 of these miRNAs had, in average, 0.5 copy per cell. Among these 185 miRNAs, only 6 of them were reduced by 50% or more, and 26 of them were upregulated by more than twofold by HDI (Figure 6). Thus, the HDI-mediated modulation of miRNA expression in B cells undergoing CSR and plasma cell differentiation is selective.

### HDI Upregulates Selected miRNAs that Target *Aicda*

**Figure 7:**
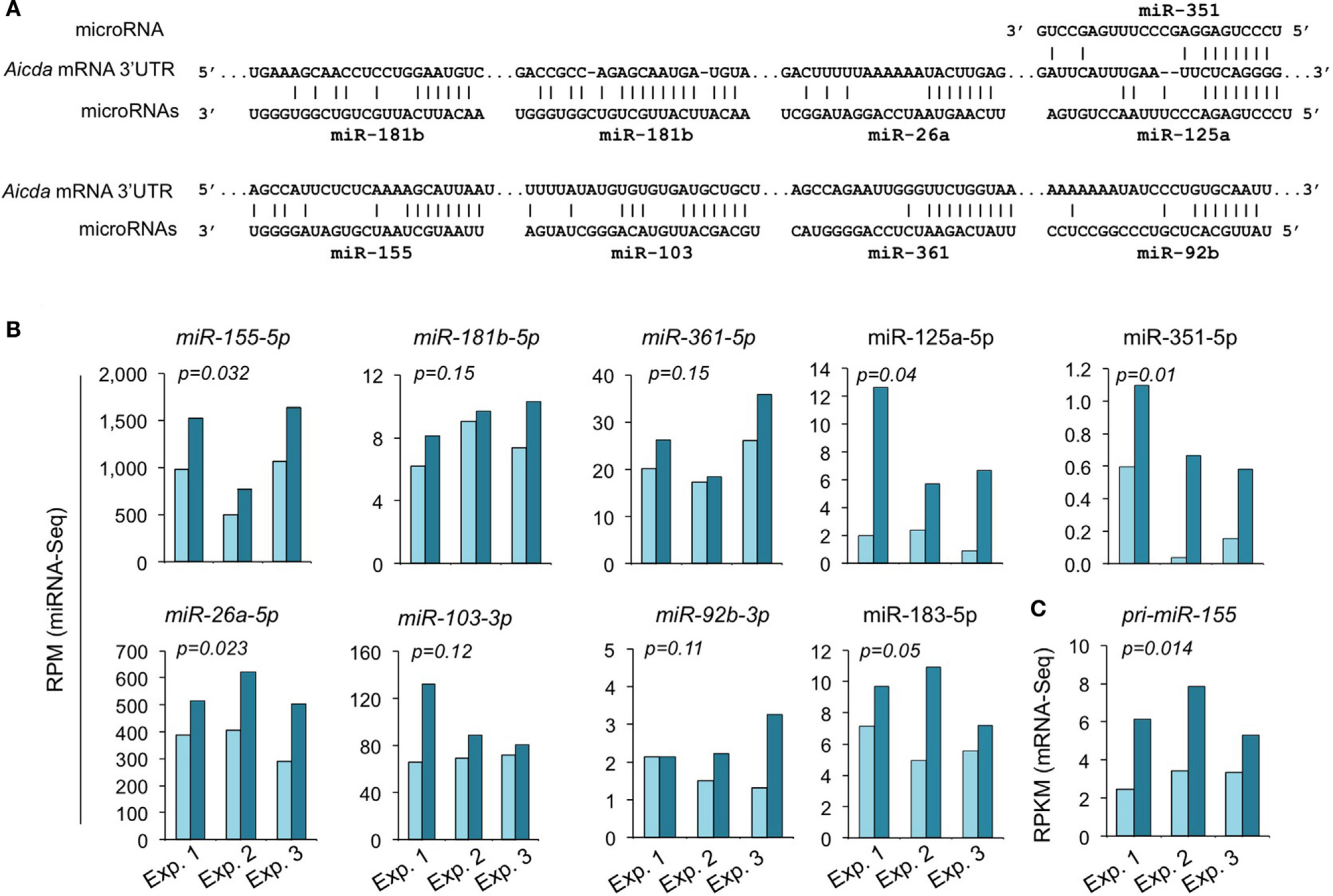
**HDI upregulates the miRNAs that target *Aicda***. **(A)** Alignment of the miRNAs and their target sites in the 3′ UTR of *Aicda* mRNAs. **(B)** Expression of miRNAs that modulate AID or are predicated to modulate AID in B cells cultured with LPS plus IL-4 in the presence of HDI (VPA, 500 μM) or nil for 60 h were analyzed by miRNA-Seq and depicted as RPM. **(C)** Primary (pri-) miRNA transcripts of miR-155 in B cells cultured for 60 h with LPS plus IL-4 in the presence of nil or HDI were analyzed by mRNA-Seq and depicted as RPKM. Data are from three independent experiments. *p* values, paired *t*-test.

We have shown by qRT-PCR that miR-155, miR-181b, and miR-361, which silence AID by targeting *Aicda* 3′ UTR, were significantly upregulated by HDI (16). The HDI-mediated upregulation of these miRNAs, particularly, miR-155 was validated by miRNA-Seq in three independent experiments (Figure 7). miR-155 targets a highly conserved site in the 3′ UTR of *Aicda* mRNA in several different species (45, 46). As shown by miRNA-Seq analysis, miR-155 is one of the most abundant miRNAs expressed in B cells after stimulation by LPS plus IL-4 (Figure 6). In such B cells, the average RPM of miR-155 was 684.9 (572.0, 981.7, and 501.0 in three independent experiments, respectively), which is more than 25 times higher than the average RPM of all the 520 miRNAs analyzed. Upon treatment with HDI, miR-155 expression was increased by more than 1.9-fold (*p* = 0.032) (Figure 7). miR-155 is encoded by the miR155 host gene *miR155HG*. *miR155HG* was originally identified as a gene that was transcriptionally activated by promoter insertion at a common retroviral integration site in B cell lymphomas and was formerly referred to as *Bic* (*B* cell *i*ntegration *c*luster) (13). Consistent with our qRT-PCR results (16), the mRNA-Seq data showed that HDI-mediated upregulation of miR-155 was associated with an increase of primary *miR-155HG* transcript (Figure 7C). In addition to the targeting sites for miR-155, miR-181b, and miR-361, the 3′ UTR of mouse *Aicda* mRNA also contains the putative target sites for miR-125a, miR-351, miR-92b, miR-26a, and miR-103 (identified by using miRNA-targeting prediction tools: TargetScan.org, miRNA.org, and miRbase.org). These miRNAs were also upregulated by HDI. Thus, HDI upregulate miRNAs that silence *Aicda* and do so by increasing the primary transcripts of host genes of these miRNAs.

### HDI Upregulate miRNAs that Target *Prdm1*

**Figure 8:**
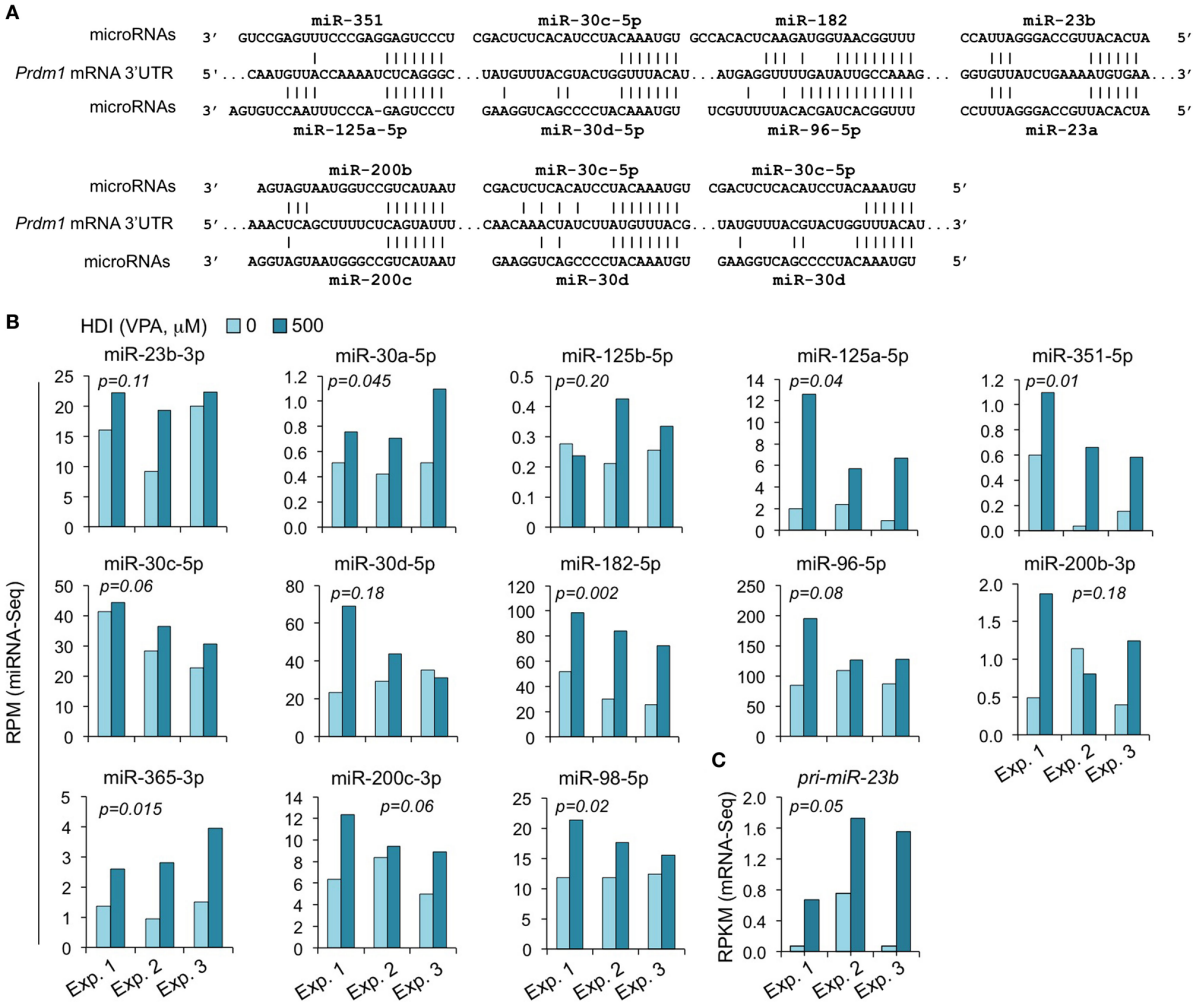
**The *Prdm1* targeting miRNAs miR-23b, miR-125a, miR-351, miR-30a/c/d, miR-182, miR-96, miR-98, miR-200b/c, and miR-365 are upregulated by HDI**. **(A)** Alignment of the 3′ UTR of *Prdm1* mRNA and the targeting miRNAs. **(B)** Expressions of the miRNAs miR-23b, miR-125a, miR-351, miR-30a/c/d, miR-182, miR-96, miR-98, miR-200b/c, and miR-365 that target or are predicted to target *Prdm1* 3′ UTR, as well as miR-183, which is expressed in a same cluster with miR-182, in B cells cultured with LPS plus IL-4 in the presence of HDI (VPA, 500 μM) or nil for 60 h were analyzed by miRNA-Seq and depicted as RPM. **(C)** Primary (pri-) miRNA transcripts of miR-23b in B cells cultured with LPS plus IL-4 in the presence of nil or HDI for 60 h were analyzed by mRNA-Seq and depicted as RPKM. Data are from three independent experiments. *p* values, paired *t*-test.

We have shown that HDI significantly downregulated *Prdm1* expression, plasma cell differentiation, and antibody/autoantibody production (16). The greatly reduced plasma cell differentiation was associated with downregulation of the Ig J chain (*IgJ*) gene, which is expressed only after B cells terminal differentiation into plasma cells (47). IgJ is incorporated into an IgM pentamer or an IgA dimer and is necessary for both the cellular and mucosal secretion of antibodies. In B cells stimulated by LPS plus IL-4 for 60 h, the average RPKM of *IgJ* mRNA was 344.6 (395.1, 175.6, and 463.2 in three independent experiments, respectively). HDI reduced *IgJ* expression (RPKM) by more than 3.3-fold to average 103.6 (77.3, 46.2, and 187.3, in three independent experiments, respectively) (Figure 2). Both human *PRDM1* and mouse *Prdm1* mRNA have a long (2,453 bp) 3′ UTR, including putative miRNA-targeting sites, which are evolutionary conserved. By using miRNA-targeting prediction tools (TargetScan.org, miRNA.org, and miRbase.org), we identified miR-125a, miR-125b, miR-96, miR-351, miR-30, miR-182, miR-23a, miR-23b, miR-200b, miR-200c, miR-33a, miR-365, let-7, miR-98, miR-24, miR-9, miR-223, and miR-133 as *PRDM1/Prdm1* targeting miRNAs in both the human and the mouse. With exception of miR-33a, miR223, miR-9, miR-24, and miR-429, whose expression level was low in activated B cells, such *Prdm1*-targeting miRNAs were significantly upregulated by HDI. We have shown by qRT-PCR that miR-23b, miR-30a, and miR-125b, which silence Blimp-1 by targeting *Prdm1* 3′ UTR, were significantly upregulated by HDI (16). The upregulation of these miRNAs was further validated in B cells by miRNA-Seq in three independent experiments (Figure 8). This likely resulted from increased primary miRNA transcripts, as suggested by the upregulation of *pri-miR-23b*.

The miR-30 family consists of five miRNAs (miR-30a, miR-30b, miR-30c, miR-30d, and miR-30e) encoded by different host genes. The miR-30 family members are similar to each other and have identical seed sequences. Like human *PRDM1* (48), the 3′ UTR of mouse *Prdm1* mRNA contains three highly conserved bindings sites complementary to the seed sequence of miR-30a and other miR-30 family members (Figure 8). All the five miR-30 miRNAs were expressed in B cells stimulated by LPS plus IL-4. The abundance of miR-30b, miR-30c, miR-30d, and miR-30e were greater than that of miR-30a (Figure 8). miR-30c was upregulated by HDI in all the three experiments, miR-30d was upregulated in two of the three experiments, while miR-30b and miR-30e were upregulated in one of the three experiments but were downregulated in the other two experiments.

miR-125 is a an evolutionarily conserved miRNA family consisting of three paralogs, including miR-125a, miR-125b-1, and miR-125b-2 (miR-125b). Recent studies have presented strong evidence for a role of the miR-125 family in the immune response. miR-125a shares the same seed sequence with miR-125b. Like miR-125b, miR-125a also potentially targets *Prdm1* in both human being and mouse, as predicted by the sequences (Figure 8). The abundance of miR-125a in B cells induced to undergo CSR and plasma cell differentiation by LPS plus IL-4 was much greater than that of miR-125b, as in all three experiments. In the presence of HDI, miR-125a expression was increased by up to 7.5-fold, perhaps suggesting a more important role of this miRNA than miR-125b in modulating *Blimp1* expression. miR-125a and miR-351 contain the same seed sequence as miR-125b, and therefore potentially target *Prdm1* 3′ UTR at the same site as miR-125b. Likewise, HDI upregulated miR-351 expression by up to 16.5-fold. miR-98 potentially target the same site as let-7 in *Prdm1* 3′ UTR. While let-7 miRNAs were not consistently altered by HDI, miR-98 was significantly upregulated by HDI in B cells in all the three experiments of CSR/plasma cell differentiation induction (*p* = 0.02).

miR-182, miR-96, and miR-183 belong to a polycistronic miRNA cluster that is located within a 4-kb area on mouse chromosome 6q. These miRNA siblings share similar seed sequences; in fact, the seed sequences of miR-96 and miR-182 are identical. As predicted using online miRNA analyze tools, TargetScan.org, miRNA.org, and miRbase.org, in both mouse and human, miR-182 and miR-96 can potentially target *Prdm1/PRDM1* 3′ UTR at the same site (Figure 8). In B cells stimulated with LPS plus IL-4, miR-182, miR-96, and miR-183 were all highly expressed. All these three miRNAs were upregulated by HDI. Because the precursors of miR-96, miR-182, and miR-183 are transcribed as a single transcript, these findings further support the contention that HDI modulate miRNA expression through regulation of their primary transcript (16). Thus, these experiments showed that HDI upregulate the miRNAs that target *Prdm1*, possibly by increasing the primary transcripts of host genes of these miRNAs.

## Discussion

Epigenetic marks/factors, such as histone posttranslational modifications, DNA methylation, and non-coding RNAs, including miRNAs, play important roles in the complex interplay between genes and environment. As we have suggested, they “interact” with genetic programs to regulate B cell functions, including CSR, SHM, and plasma cell differentiation, thereby informing the antibody response (1, 2, 7, 49). Epigenetic dysregulation can result in aberrant antibody responses and compound genetic susceptibility to mediate autoimmunity (7, 49). We have recently shown that HDI epigenetic modulators inhibited CSR, SHM, and plasma cell differentiation by modulating intrinsic B cell mechanisms (16). HDI repressed AID and Blimp-1 expression in human and mouse B cells by upregulating selected miRNAs that silenced *AICDA/Aicda* and *PRDM1/Prdm1* mRNAs, as demonstrated by multiple qRT-PCRs. In this study, we performed high throughput miRNA-Seq and mRNA-Seq to further define the HDI-mediated modulation of miRNA and gene expression. We showed here that HDI selectively upregulated miRNAs involved in targeting and modulating genes whose expressions are critical for B cells to undergo CSR and plasma cell differentiation. The selective upregulation of miRNAs and mRNAs by HDI was emphasized by unchanged expression of miRNAs, which are not known to regulate *Aicda* or *Prdm1*, the master genes for CSR/SHM or plasma cell differentiation, and unchanged expression of the genes that are involved in regulation, targeting, or DNA repair processes in CSR/SHM, as well as the genes of epigenetic regulators and the factors that are important for cell signaling and apoptosis. Consistent with the notion that HDI downregulate mRNA expression by upregulating selective miRNAs, HDI slightly reduced average RPKM of overall mRNA, in association with a slightly increased overall miRNA expression. This study further extends our previous findings and outlines more precisely epigenetic mechanisms that are critical to the B cell differentiation processes that underpin antibody and autoantibody responses.

In spite of the broad distribution of HDACs in chromatin, our findings showed that HDI-mediated modulation of miRNA and mRNA expression is very selective. Upon exposure to HDI, 18 genes were upregulated by more than twofold in B cells induced to undergo CSR and plasma cells differentiation. These genes, included *Zbtb20* (Zinc finger and BTB domain-containing protein 20), a Bcl6 homolog, which is highly expressed in activated B cells and memory B cells and has been shown to regulate long-term antibody production through a B cell-intrinsic mechanism (50, 51), and *Syt11* (synaptotagmin-11), which is specifically expressed in memory B cells (52), suggesting that HDI can modulate the memory B cell response. Sixteen genes were downregulated by HDI by more than 50%. More than half of these downregulated genes, including *Bhlha15, Rhob, Fkbp11, Ppapdc1b, Rcbtb2, Ly6c2, Txndc5*, and *IgJ*, are preferentially expressed in plasma cells rather than naïve, germinal center, or memory B cell (52) (http://www.ncbi.nlm.nih.gov/sites/GDSbrowser?acc=GDS1695). This may imply that, in addition to the inhibition of AID and Blimp-1 expression, alteration of the expression of other genes that are involved in B cell differentiation could also contribute to HDI-mediated modulation of the antibody response. Alternatively, the reduction of these “plasma cell specific” genes may simply result from the inhibition of plasma cells differentiation by HDI, further supporting the selectivity of HDI-mediated modulation of mRNA expression. HDI modulated CSR, SHM, and plasma cells differentiation, and, therefore, the antibody response mainly through downregulation of *Aicda, Prdm1*, and *Xbp1*. However, the HDI-mediated downregulation of *Cxcr1*, which is preferentially expressed in IgG1^+^ memory B cells and promotes IgG1 autoantibody production (25, 26), and *Saa3*, which can interact with Tlr4 and induce Tlr4-mediated NF-κB activation (27), suggest that downregulation of these elements can contribute to HDI-mediated modulation of antibody response. The reduction of *Cxcr3, Fut1*, and *Rhobtb1* expression was associated with an increased expression of miR-148b, miR-125a, and miR-182, which target *Cxcr3*, *Fut1*, and *Rhobtb1* mRNAs, respectively, suggesting that, in addition to *Aicda* and *Prdm1*, which are already downregulated by HDI, other genes can also be downregulated by HDI through upregulation of their targeting miRNAs. This does not, however, exclude the possibility that the HDI-mediated reduction of gene expression may at least partially result from altered expression or activation of other B cell factors.

A total of 18 HDACs, which are not functionally redundant, have been identified in human beings and mice (12). These 18 HDACs are grouped into four classes based on their function and sequence similarity (12). Classes I, II, and IV consist of 11 HDACs that require zinc as a cofactor. Class I includes HDAC1, HDAC2, HDAC3, and HDAC8, which display homology to yeast RPD3; Class IIa includes HDAC4, HDAC5, HDAC7, and HDAC9, which display homology to yeast HDA1; Class IIb includes HDAC6 and HDAC10, which contain two catalytic sites. Class IV includes only HDAC11, which displays conserved residues in its catalytic center, shared by both class I and class II HDACs. Class III HDACs or Sirtuins (Sirt1-7) display homology to yeast Sir2, retain NAD-dependent catalytic sites and share some functions with the classical HDACs. Unlike the classes I, II, and IV HDACs, Sirtuins are not zinc dependent and cannot be inhibited by conventional HDI, such as VPA and butyrate. They may function differently from class I/II HDACs in the regulation of the antibody response. Indeed, activation of Sirt1 by resveratrol has been shown to lead to a reduced production of IgG1 and IgG2a in pristane-induced lupus mice (53) as well as antigen-specific IgE in OVA-immunized mice (54).

Our findings showed that HDI selectively downregulated the expression of those genes that are central to CSR and plasma cell differentiation processes, that is, *Aicda* encoding AID cytidine deaminase and *Prdm1* encoding Blimp-1. They did not, however, downregulate the expression of 14-3-3 adaptors, which, as we showed, are upregulated by the stimuli that induce CSR and are important for AID targeting to S regions in CSR (2, 29, 30, 32). Rab7, an effective multifunctional regulator of autophagy, which activates the canonical NF-κB pathway to induce AID expression (35), was also unchanged by HDI. In addition, DNA repair factors, such as Ung and TLS DNA polymerases, which play important roles for CSR and SHM, were also not altered by HDI. Further, epigenetic regulators HATs and HDACs, and Tet proteins, genes that are important in cell apoptosis, such as *Mcl1, Bcl2*, and *Bcl2l1*, TLRs, and genes involved in NF-κB signaling also remained unchanged in B cells induced to undergo CSR. The selective regulation of gene expression by HDI was consistent with what have been reported in other type of cells (55–58), and the level of changes in transcription was associated with the type and doses of HDI used and the time of culture (23). Although most of the available HDI do not have a high HDAC isoform specificity, SCFA HDI have been suggested to display significant selectivity for different HDACs. For example, VPA targets class I HDACs, particularly, HDAC1 and HDAC2, and, less effectively, class IIa HDACs, butyrate targets class I, mainly HDAC1, and, less effectively, other members of class I and class IIa HDACs. Like HATs, HDACs do not directly bind to DNA, rather, they interact with DNA through multi-protein complexes that include coactivators and corepressors, the role and composition of which are often cell type-specific (59). HDAC-associated proteins would specify the selectivity of HDI, which display different affinities for different HDAC/cofactor complexes. HDI with diverse chemical properties target different HDACs and HDAC/cofactor complexes, thereby regulating gene expression in a locus- and cell type-specific fashion. Thus, the different HDAC-associated proteins and the different HDAC/cofactor complexes would provide the mechanistic underpinning for the selectivity of HDI for specific B cell differentiation genes, as we have shown here.

*PRDM1/Prdm1* mRNA contains a long (2,453 bp) conserved 3′ UTR, which comprises putative target sites for multiple miRNAs. In addition to miR-23b, miR-30a, and miR-125b, which, as we showed by qRT-PCR and miRNA-Seq, are upregulated by HDI, several other putative *Prdm1* targeting miRNAs, including miR-125a, miR-96, miR-351, miR-30c, miR-182, miR-23a, miR-200b, miR-200c, miR-365, let-7, miR-98, and miR-133, were also significantly increased by HDI. miR-182 has been identified as the miRNA induced at a high level in B cells stimulated to undergo CSR (60); however, deficiency of this miRNA did not significantly alter the titers of total serum IgM, IgG1, IgG2a, IgG2b, IgG3, IgA, and IgE, and NP-binding IgG1 in mice immunized with NP-CGG (60). miR-182 is a member of the miR-183~182 cluster which includes miR-96, miR-182, and miR-183. Like miR-182, miR-96, which, based on its sequence, could target all putative miR-182 targeting sites, is also highly expressed by B cells induced to undergo CSR and plasma cell differentiation (Figure 8), would compensate the function of miR-182. miR-183, another member of miR-183~182 cluster, was also upregulated by HDI. This together with our finding that all members of miR-99b~let-7e~125a cluster were increased by HDI further confirm that HDI modulation of miRNA expression occurs through modulation of miRNA primary transcript. There is no conserved miRNA-targeting site identified in the 3′ UTR of *Xbp1* mRNA. The 3′ UTR of mouse *Xbp1* mRNA contains several putative target sites for miR-199, miR-299, miR-433, miR-221, and miR-490. None of these miRNAs, however, were increased by HDI, supporting the contention that the HDI-mediated reduction of *Xbp1* resulted from decreased *Prdm1* expression (16).

These findings demonstrated that HDI modulate CSR, SHM, and plasma cell differentiation, and, therefore, antibody responses by downregulating *Aicda* and *Prdm1* expression through upregulation of targeting miRNAs. By significantly extending our recent findings (16), it provides further and strong evidence that HDI, including those commonly known as “pan-HDI,” can effectively modulate a restricted spectrum of miRNAs and, thereby, mRNAs in B cells induced to undergo CSR and plasma cell differentiation. This results from HDACs existing in the unique contexts of HDAC/cofactor complexes, as occurring in B lymphocytes, particularly when in an activated state. The fine specificity of the mechanisms of miRNA/mRNA regulation revealed here was emphasized by the failure of HDI to modulate a variety of other mRNAs encoding elements that participate in but do not initiate the processes of events that leads to CSR. Finally, our studies also provide mechanistic insights into epigenetic mechanisms that directly modulate B cell-intrinsic functions in the immune response, thereby offering new clues for further therapeutic approaches, as specifically targeted to B cells.

## Conflict of Interest Statement

The authors declare that the research was conducted in the absence of any commercial or financial relationships that could be construed as a potential conflict of interest.
